# Theoretical Modeling and Experimental Validation of Contact Pressure in the Solid Rocket Motor Thermal Insulation Winding Process

**DOI:** 10.3390/ma19173599

**Published:** 2026-08-24

**Authors:** Weichao Zhang, Zengxuan Hou

**Affiliations:** State Key Laboratory of High-Performance Precision Manufacturing, School of Mechanical Engineering, Dalian University of Technology, Dalian 116024, China; zwc@mail.dlut.edu.cn

**Keywords:** solid rocket motor, thermal insulation winding, contact pressure distribution, semi-empirical modeling, elastic foundation model, thin elastic layer

## Abstract

In the thermal insulation winding process of solid rocket motors, the roller-tape contact pressure is a critical factor determining bonding quality. However, accurately predicting this pressure is challenging due to the complex three-dimensional contact involving a thin, nearly incompressible rubber tape and an elliptical concave press roller. This paper proposes a theoretical model extending the classical elastic foundation model by incorporating correction strategies to account for material incompressibility and geometric confinement. A finite element (FE) model was developed to simulate the contact and verified against Hertz theory. Two key parameters of the theoretical model were calibrated using the FE results and justified through a parametric study on Poisson’s ratio and a theoretical analysis of the contact half-width. The theoretical predictions of deformation, contact pressure distribution, and pressing force agree well with the FE results under different applied displacements and mandrel radii without parameter recalibration, demonstrating the model’s generality. Experimental validation employing hybrid inverse analysis confirms the model’s global accuracy, yielding <11% relative error between the predicted and measured pressing forces. This study establishes a theoretical foundation for pressure control in the winding process and provides insights into contact problems for thin elastic layers with high Poisson’s ratios (≥0.45).

## 1. Introduction

**Figure 1:**
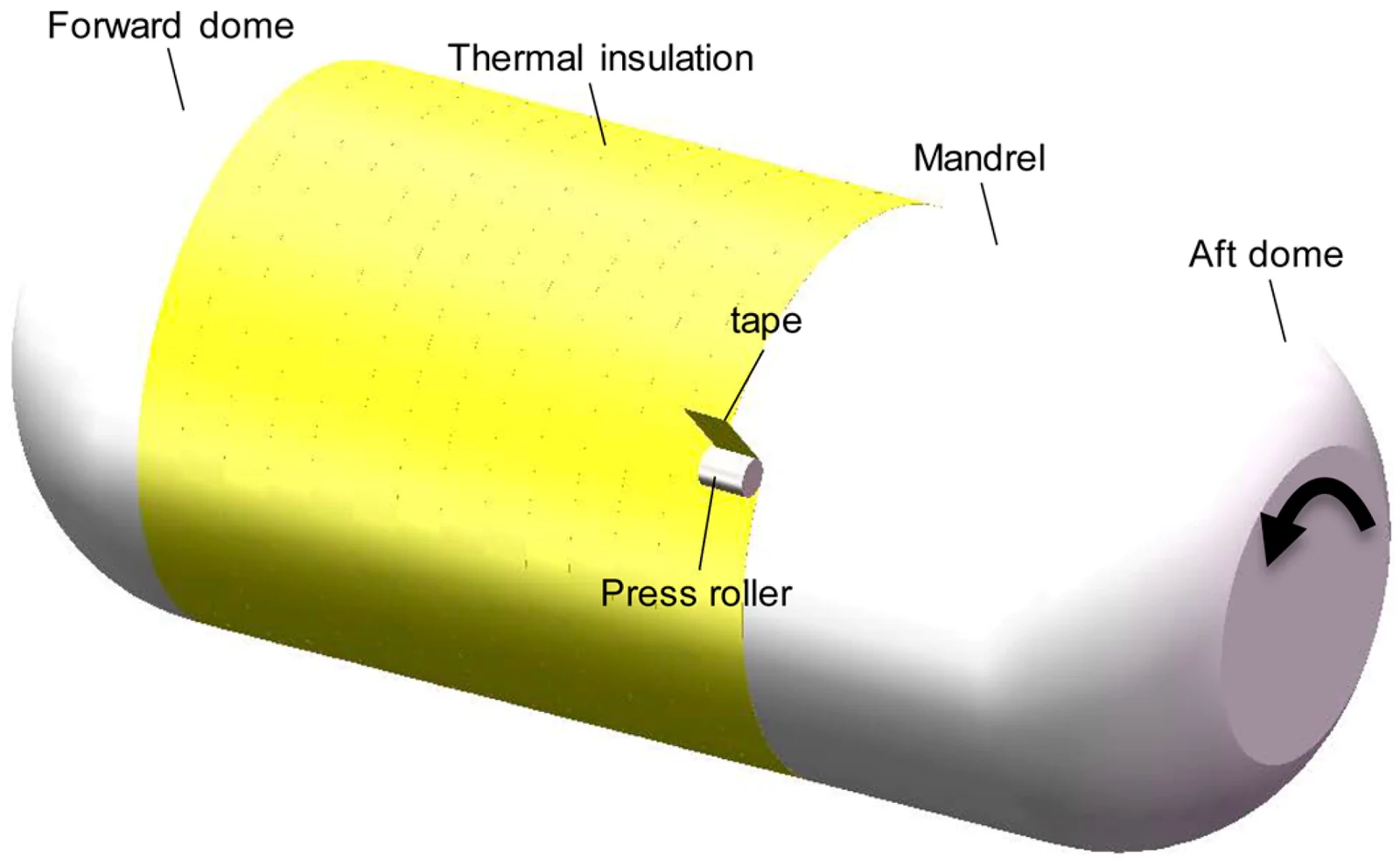
Schematic of the thermal insulation winding process.

Internal thermal insulation serves as a critical heat barrier in solid rocket motors [1]. While various fabrication methods for the thermal insulation exist, including manual lay-up and molding, the winding process [2,3] provides distinct advantages for manufacturing large-scale motors by significantly reducing cycle time and ensuring consistent quality. In this technique, each tape is continuously wound onto a rotating mandrel [4] (Figure 1). A key component of this process is the press roller, which applies pressure to bond the incoming tape onto the substrate (mandrel or underlying layers).

The roller-tape contact pressure plays a crucial role in determining the bonding quality of the thermal insulation. Insufficient pressure is a primary cause of defects such as edge lifting. Therefore, it is essential to study the contact pressure distribution to ensure the required pressure.

Finite element (FE) models are widely employed to simulate and analyze contact pressure distribution. For example, Zhang et al. [4] developed an FE model of the roller-tape contact in the thermal insulation winding process. Through the analysis of contact pressure distribution, an optimization strategy for the press roller was proposed. Bakhshi et al. [5] utilized FE simulation to examine the effects of stiffness and architecture of different press rollers on contact pressure distribution in automated fiber placement (AFP). Based on the simulation results, the impact of the rollers on the layup quality was analyzed.

In theoretical terms, Hertz theory is commonly used to analyze the contact pressure distribution between elastic solids. Kang [6] utilized this theory to establish a contact model for prepreg tape cylinder winding. Oromiehie et al. [7] applied Hertz theory to calculate the pressure between the roller and the thermoplastic composite tape in AFP. However, Hertz theory is based on the elastic half-space assumption [8]. Given that the tape layers are bonded to the mold, this can be considered a fixed boundary condition [7,9]. Moreover, the tape is relatively thin, and when the size of the contact area is comparable to or larger than the total thickness of the layers, this fixed boundary condition imposes geometric confinement on the deformation field, which can affect the contact pressure distribution [10,11]. Consequently, the applicability of Hertz theory in analyzing the pressure distribution may be limited.

Jiang et al. [12] developed a theoretical model for the pressure distribution on irregularly curved molds in AFP by investigating the deformation and stress at the roller–prepreg interface. Miao et al. [13,14] also proposed a theoretical model for the roller–prepreg contact on irregularly curved surfaces in AFP, based on the pressure–deformation relationships of the two contact materials and Hertz theory. However, both models do not account for the geometric confinement induced by the fixed boundary condition (where the tape layers are bonded to the mold) on the contact pressure distribution.

Research on contact problems for thin elastic layers has overcome the limitations of the elastic half-space assumption. Thin elastic layers coating another more rigid substrate are encountered in various scenarios, such as rubber-covered rollers [15] and cartilage layers attached to bones [16]. There has been considerable research on contact problems for thin elastic layers. Fourier integral transforms can be conveniently employed to formulate these problems [11]. Meijers [17] derived asymptotic solutions for the contact problem between a rigid cylinder and an elastic layer bonded to a rigid substrate. Greenwood et al. [18] developed an efficient and accurate method for determining the Green’s function to solve contact problems involving a rigid cylinder indenting an elastic layer either bonded or unbonded to a rigid substrate.

The elastic foundation model (EFM) is often used to solve contact problems for thin elastic layers. Pandy et al. [19] utilized the EFM to calculate the pressure distributions in the tibiofemoral joint, treating the layers of cartilage as a single equivalent layer on a rigid foundation. Li et al. [20] proposed a double elastic layer EFM to calculate the pressure distribution of a bimetallic bearing.

However, the tape material used in this study is uncured ethylene propylene diene monomer (EPDM) rubber, which exhibits near-incompressibility, with a Poisson’s ratio of approximately 0.5 [21]. In contact problems for thin elastic layers, when the material has a high Poisson’s ratio (≥0.45), the EFM yields unreliable results [22]. Although Meijers [17] and Greenwood et al. [18] investigated contact problems for thin elastic layers with high Poisson’s ratios, their analyses were limited to the plane strain conditions of line contact.

In the thermal insulation winding process, the press roller contacts the rubber tape in a three-dimensional, surface-to-surface configuration, which cannot be accurately described by a plane strain model. Moreover, the press roller employed in this study features an elliptical concave surface, further complicating the modeling of this contact. While the geometric advantage of this elliptical concave press roller (ECPR) has been demonstrated in a recent study [4], that work focused purely on structural optimization relying on time-consuming FE simulations. Consequently, the heavy reliance on such cumbersome FE setups has become a major practical bottleneck for rapid pressure prediction. To the best of our knowledge, such three-dimensional contact problems for thin elastic layers involving the high Poisson’s ratio rubber tape and the ECPR in the thermal insulation winding process remain underexplored and challenging.

Therefore, the primary purpose of this study is to develop a theoretical model to predict the contact pressure distribution between the ECPR and the rubber tape. The novelty of this model lies in extending the classical EFM by incorporating correction strategies to account for material incompressibility and geometric confinement, thereby addressing the inherent limitations of the classical EFM. The proposed model is cross-validated by both numerical and experimental methods. This work provides a theoretical basis for pressure control in the thermal insulation winding process.

## 2. Theoretical Modeling

### 2.1. Geometric Characteristics of the ECPR

The thermal insulation was fabricated using the molding process for the forward and aft dome sections and the winding process for the cylindrical body, the latter of which comprised two wound layers. Defects such as edge lifting may occur during the winding process when a cylindrical press roller is used. This is primarily due to the inconsistent distance between the roller and the mandrel (or thermal insulation) along the tape width. Specifically, the increased distance at the tape edges leads to insufficient pressure, thereby compromising the overall winding quality. To ensure the winding quality of the thermal insulation, an ECPR is employed [4].

**Figure 2:**
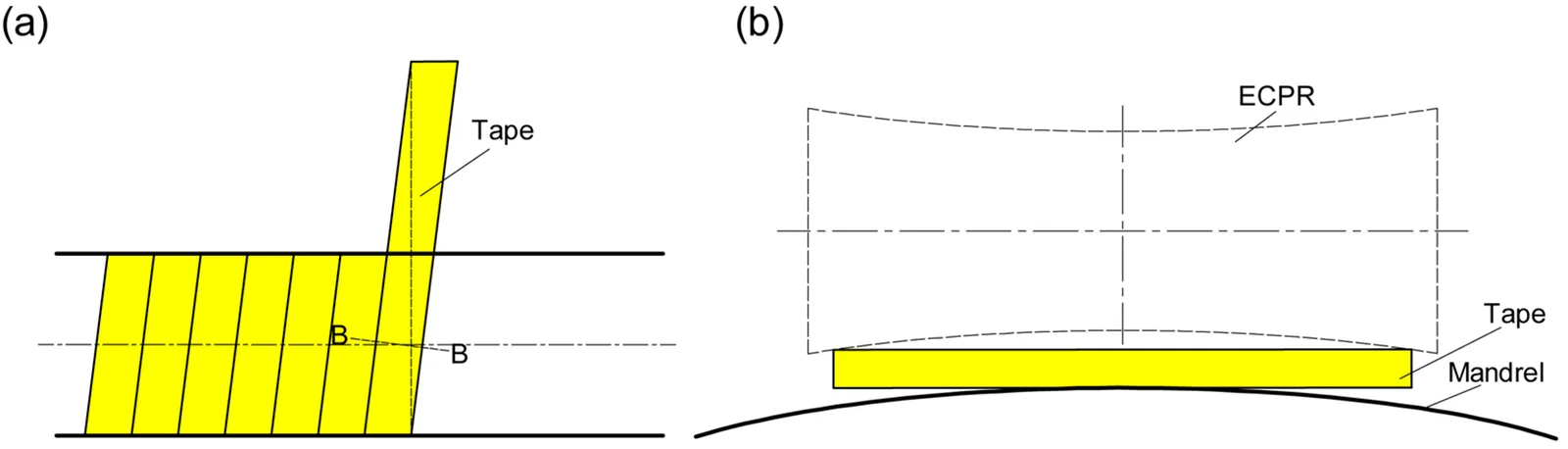
Geometric generation of the elliptical concave press roller (ECPR): (**a**) unfolded view of the tape along the winding path; (**b**) geometry of the roller in longitudinal section B-B, which is perpendicular to the winding direction and passes through the roller center.

As detailed in [4], the generatrix of the ECPR is designed by translating the profile of the smallest-radius mandrel (shown in Figure 2b), ensuring optimal conformability during contact. Replacing the cylindrical press roller with the ECPR significantly enhances pressure distribution uniformity on the smallest-radius mandrel. Furthermore, this design reduces the risk of edge lifting by increasing contact pressure at the tape edges.

### 2.2. Formulation of the Theoretical Model

**Figure 3:**
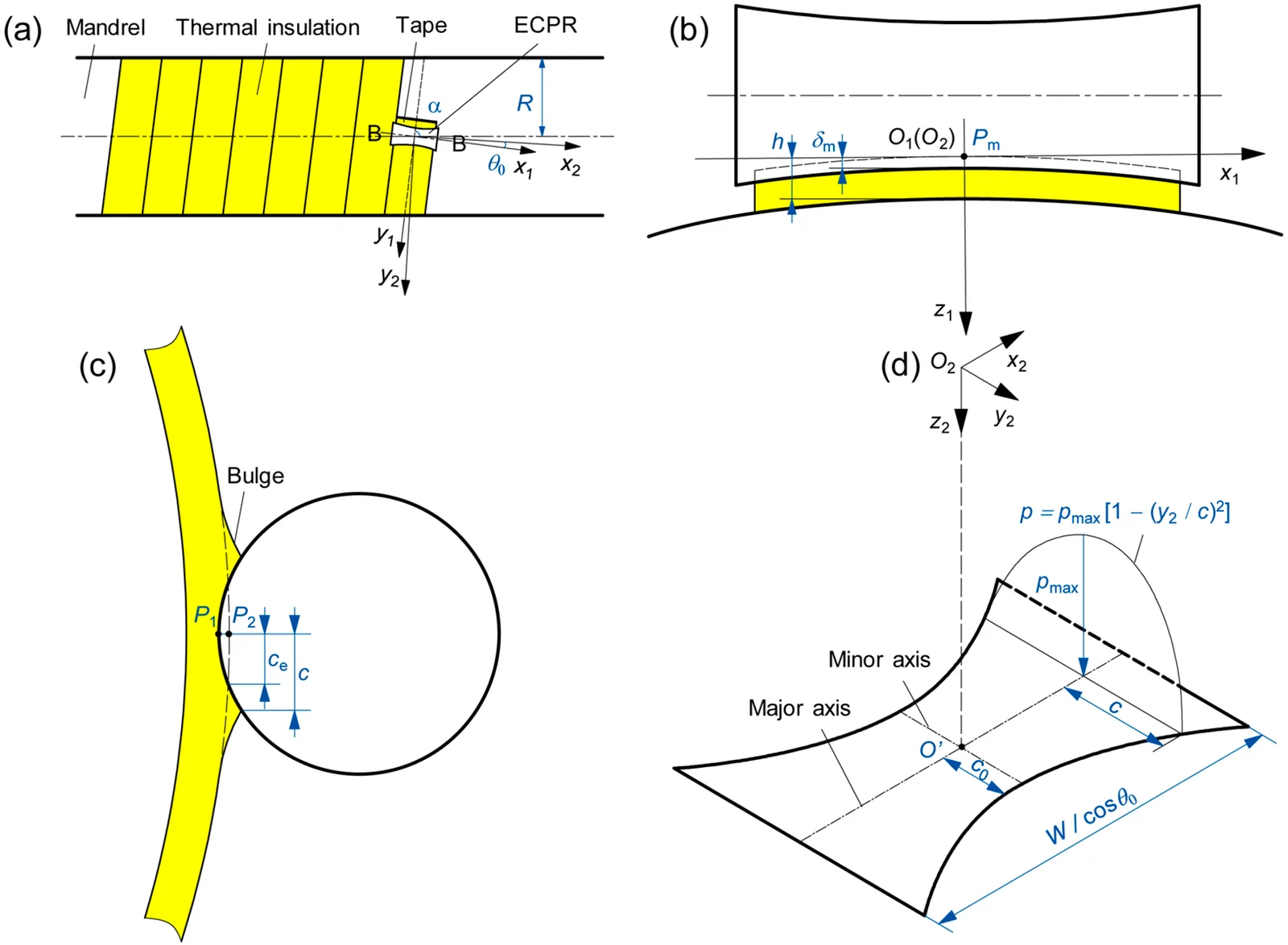
Details of the ECPR–tape contact: (**a**) hoop winding schematic; (**b**) interaction in longitudinal section B-B; (**c**) view parallel to the *y*_2_*z*_2_ plane; (**d**) idealized contact pressure distribution.

In the winding process, pressure is applied by pressing the ECPR into the tape to a predetermined depth, as shown in Figure 3a–c.

To analyze the pressure distribution, spatial rectangular coordinate systems are established (Figure 3a,b). The origin of the coordinate system {*O*_1_} is defined at the initial position of point *P*_m_, which is the midpoint of the tape’s top edge in the longitudinal section B-B. The *y*_1_-axis is tangential to the helical winding path at *P*_m_, and the *z*_1_-axis is normal to the surface at *P*_m_. The direction of the *x*_1_-axis is determined using the right-hand rule; the axis of the press roller is parallel to the *x*_1_-axis. The coordinate system {*O*_2_} is obtained by rotating the coordinate system {*O*_1_} clockwise about the *z*_1_-axis by an angle *θ*_0_.

The present study focuses exclusively on scenarios involving elastic deformation within the contact bodies, assuming frictionless interfaces. To obtain an approximate solution for the contact pressure distribution, we make the following assumptions:

(1) The press roller and the mandrel are assumed to be rigid bodies, given that their elastic moduli are substantially higher than that of the tape.
(2) The combination of the mandrel and the thermal insulation is modeled as a composite cylinder. Its radius is equal to the sum of the mandrel radius and the thermal insulation thickness. The ECPR–tape contact area is idealized, as shown in Figure 3d. It is symmetric about both its major and minor axes, which are parallel to the *x*_2_-axis and the *y*_2_-axis, respectively. The angle *θ*_0_ between the major axis and the *x*_1_-axis is calculated using Equation (1).

(1) $$\theta_{0}=\frac{1}{2}\arctan\frac{r_{0}\sin\left(2\alpha \right)}{\left(R+t\right)-r_{0}\cos\left(2\alpha \right)}$$

(2) $$\cos\alpha =\frac{W}{2\pi \left(R+t-h\right)}$$

where *r*_0_ is the radius of the central cross-section of the ECPR, *α* is the winding angle, *W* is the width of the tape, *R* is the radius of the winding mandrel, *t* is the thickness of the thermal insulation, and *h* is the thickness of the tape.

(3) As shown in Figure 3d, the contact pressure distribution in any section parallel to the *y*_2_*z*_2_ plane follows the parabolic profile described by:

(3) $$p=p_{\max}\left[1-{\left(\frac{y_{2}}{c}\right)}^{2}\right]$$

where *p*_max_ is the maximum contact pressure in the section, and *p* is the contact pressure along the contact width 2*c*.

The rationale for proposing Assumption (2) is detailed below.

For a cylindrical press roller with radius *r* employed in the configuration shown in Figure 3a, the contact area between the press roller and the composite cylinder is elliptical, as predicted by both Hertz theory and the EFM [10]. Although the ellipse dimensions differ between the two methods, their major axes have the same orientation. The major axis forms an angle *θ* with the press roller axis. The formula for this angle is given by [10]:

(4) $$\theta =\frac{1}{2}\arctan\frac{r\sin\left(2\alpha \right)}{\left(R+t\right)-r\cos\left(2\alpha \right)}$$

The partial derivative of *θ* with respect to *r* is expressed as:

(5) $$\frac{\partial \theta }{\partial r}=\frac{\left(R+t\right)\sin\left(2\alpha \right)}{2{\left(R+t\right)}^{2}+2r^{2}-4\left(R+t\right)r\cos\left(2\alpha \right)}$$

Given a small change Δ*r* in the radius of the cylindrical press roller, the relative change in the angle *θ* is given by:

(6) $$\frac{\Delta \theta }{\theta }\approx \frac{\Delta r\partial \theta }{\theta \partial r}$$

where Δ*θ* is the change in the angle *θ*.

In this study, the length of the ECPR is 102 mm, and its central cross-sectional radius is 40 mm. The radius of the smallest mandrel is 240 mm. The thickness of the tape is 0.8 mm, and its width is 100 mm. Under the constraints (7), the maximum value of Δ*θ*/*θ* is less than 0.06%.

(7) $$\left\{\begin{array}{c} r_{0}\leq r\leq r_{e}\\ R\geq R_{0}\\ t\geq h \end{array}\right.$$

where *r*_e_ is the end-face radius of the ECPR, and *R*_0_ is the radius of the smallest mandrel.

Thus, when *R* and *t* are constant, and the radius *r* of the cylindrical press roller varies between *r*_0_ and *r*_e_, the angle *θ* remains approximately constant relative to its value at *r* = *r*_0_, with an increase of less than 0.06% over this range. Therefore, when the ECPR is in use, the major axis can be assumed to exist. This major axis forms the angle *θ*_0_ with the *x*_1_-axis and is parallel to the *x*_2_-axis.

Additionally, experimental results from [12,13] indicate that the contact area between a cylindrical press roller and prepreg tapes on an irregular curved mold is approximately symmetric at different contact points. In this study, if the ECPR is considered as a mold and the composite cylinder as a cylindrical press roller, it can be inferred from [12,13] that the contact area between the cylindrical press roller and the mold is also approximately symmetric.

Therefore, the ECPR–tape contact area is idealized. Figure 3d shows the shape of the contact area. The contact area is symmetric about both the major and minor axes.

The theoretical model is established as follows:

(1) Formulate the surface equations of the ECPR and the composite cylinder.

The surface equation of the ECPR in the coordinate system {*O*_1_} can be expressed as:

(8) $$\left\{\begin{array}{c} y_{1}^{2}+{\left(z_{1e}-s+r_{0}\right)}^{2}={\left(-\sqrt{R_{0}^{2}-x_{1}^{2}\cos^{2}\alpha_{0}}+r_{0}+R_{0}\right)}^{2}\\ \frac{-L}{2}\leq x_{1}\leq \frac{L}{2} \end{array}\right.$$

(9) $$\cos\alpha_{0}=\frac{W}{2\pi R_{0}}$$

where *s* is the applied displacement, and *L* is the length of the ECPR. Correspondingly, the surface equation of the composite cylinder in the coordinate system {*O*_1_} is given by:

(10) $${\left(x_{1}\cos\alpha +y_{1}\sin\alpha \right)}^{2}+{\left(z_{1c}-R-t\right)}^{2}={\left(R+t\right)}^{2}$$

(2) Derive the maximum contact pressure in any section parallel to the *y*_2_*z*_2_ plane.

According to Equation (8), we obtain:

(11) $$z_{1e}=\sqrt{{\left(-\sqrt{R_{0}^{2}-x_{1}^{2}\cos^{2}\alpha_{0}}+r_{0}+R_{0}\right)}^{2}-y_{1}^{2}}-r_{0}+s$$

Similarly, according to Equation (10), it follows that:

(12) $$z_{1c}=-\sqrt{{\left(R+t\right)}^{2}-{\left(x_{1}\cos\alpha +y_{1}\sin\alpha \right)}^{2}}+R+t$$

Since the coordinate system {*O*_2_} is obtained by rotating the coordinate system {*O*_1_} about the *z*_1_-axis by the angle *θ*_0_, the following coordinate transformation applies:

(13) $$\left\{\begin{array}{c} x_{1}=x_{2}\cos\theta_{0}+y_{2}\sin\theta_{0}\\ y_{1}=-x_{2}\sin\theta_{0}+y_{2}\cos\theta_{0}\\ z_{1}=z_{2} \end{array}\right.$$

Substituting Equation (13) into Equations (11) and (12) yields the surface equations *z*_2e_(*x*_2_, *y*_2_) and *z*_2c_(*x*_2_, *y*_2_), respectively.

Based on the EFM, the maximum normal displacement *δ*_max_ of the thermal insulation in any section parallel to the *y*_2_*z*_2_ plane and intersecting the contact area is expressed as:

(14) $$\delta_{\max}=z_{2e}\left(x_{2},0\right)-z_{2c}\left(x_{2},0\right)$$

The maximum contact pressure *p*_max_ in such a section is given by [23]:

(15) $$p_{\max}=K_{c}\frac{\delta_{\max}}{t}$$

where *K*_c_ is the corrected elastic modulus of the foundation, which is to be determined by other methods presented in Section 3.3.

(3) Determine the contact half-width *c*.

The radii of curvature at points *P*_1_ and *P*_2_ in Figure 3c, denoted as *R*_1_ and *R*_2_, respectively, are calculated as follows:

(16) $$R_{1}=\frac{{\left[1+{\left(\frac{\partial z_{2e}}{\partial y_{2}}\right)}^{2}\right]}^{\frac{3}{2}}}{\left|\frac{\partial^{2}z_{2e}}{\partial y_{2}^{2}}\right|}$$

(17) $$R_{2}=\frac{{\left[1+{\left(\frac{\partial z_{2c}}{\partial y_{2}}\right)}^{2}\right]}^{\frac{3}{2}}}{\left|\frac{\partial^{2}z_{2c}}{\partial y_{2}^{2}}\right|}$$

The normal displacement *δ* of the thermal insulation in any section parallel to the *y*_2_*z*_2_ plane and intersecting the contact area is as follows [10]:

(18) $$\delta =\delta_{\max}-\frac{1}{2}\left[\frac{1}{R_{1}}+\frac{1}{R_{2}}\right]y_{2}^{2}$$

Thus, in such a section, the contact half-width *c*_e_ is given by:

(19) $$c_{e}=\sqrt{2\delta_{\max}{\left[\frac{1}{R_{1}}+\frac{1}{R_{2}}\right]}^{\left(-1\right)}}$$

Since the thermal insulation is composed of uncured EPDM rubber, it exhibits near-incompressibility. When the press roller is pressed into the thermal insulation to a certain depth, the material displaced from under the roller will bulge upwards; hence, the edge of the contact area is not coincident with the point where *δ* = 0 [11]. Consequently, the contact half-width *c* will be larger than the contact half-width *c*_e_ predicted by the EFM, as shown in Figure 3c. A linear relationship between these quantities is assumed and can be expressed as follows:

(20) $$c=\lambda c_{e}$$

where *λ* denotes the contact half-width correction factor, with *λ* > 1. Its value is to be determined by other methods presented in Section 3.4.

(4) Establish the deformation and contact pressure distributions.

The deformation distribution of the thermal insulation in the *z*_2_ direction within the contact area is given by [24]:

(21) $$\left\{\begin{array}{c} \delta =\delta_{\max}\left[1-{\left(\frac{y_{2}}{c_{e}}\right)}^{2}\right]\\ -c\leq y_{2}\leq c \end{array}\right.$$

When |*y*_2_| ≤ *c*_e_, *δ* ≥ 0, indicating that the thermal insulation in this region of the contact area is compressed; when *c*_e_ < |*y*_2_| ≤ *c*, *δ* < 0, indicating that it bulges.

Based on Assumption (3), the contact pressure distribution is formulated as:

(22) $$p=\frac{K_{c}\delta_{\max}}{t}\left[1-{\left(\frac{y_{2}}{\lambda c_{e}}\right)}^{2}\right]$$

(5) Integrate the pressure distribution to obtain the pressing force *F*.

The pressing force *F* can be calculated as follows:

(23) $$F=4\iint_{D}\frac{K_{c}\delta_{\max}}{t}\left[1-{\left(\frac{y_{2}}{\lambda c_{e}}\right)}^{2}\right]dx_{2}dy_{2}$$

(24) $$D:\left\{\begin{array}{c} 0\leq x_{2}\leq \frac{W}{2\cos\theta_{0}}\\ 0\leq y_{2}\leq c \end{array}\right.$$

## 3. Finite Element Model

### 3.1. Simulation Setup

**Figure 4:**
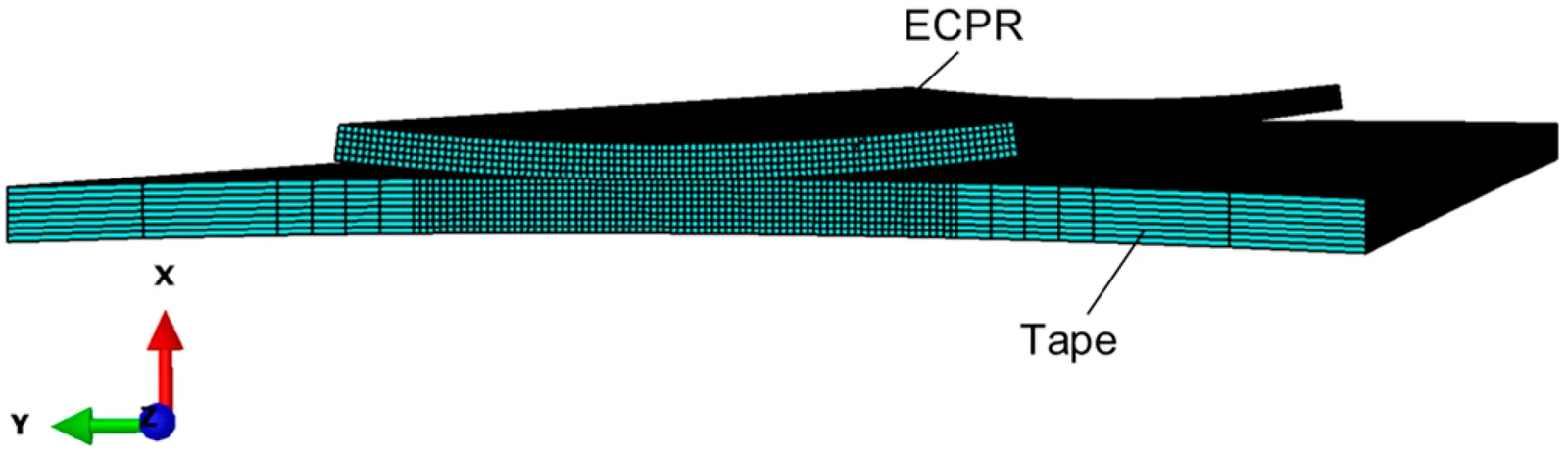
FE model setup for the ECPR–tape contact.

Following the modeling procedure established in [4], FE analysis was conducted using Abaqus/CAE to simulate the contact interaction between the ECPR and the tape. Figure 4 shows the FE model with a thermal insulation thickness of 0.8 mm (one layer). The geometric parameters of each component are consistent with those specified in Section 2.2. To reduce computational costs, the mandrel was excluded from the model. Furthermore, the press roller was modeled as a rigid, waisted annular sector (0.5 mm thickness). Given the roller’s significantly higher stiffness relative to the tape, these modeling simplifications are considered reasonable [24].

The elastic properties of the mandrel, ECPR, and tape are summarized in Table 1. In the winding process, the compressive deformation of the tape is restricted to a low-strain range (<10%). Since uncured rubber behaves linearly within this range [21,25,26] and exhibits characteristics of near-incompressibility, the tape was assigned a constant elastic modulus of 0.5 MPa [27] and a Poisson’s ratio of 0.49 [28,29]. C3D8IH and C3D8I elements were employed to model the tape and the press roller, respectively.

The press roller was constrained to translate exclusively along the *x*-axis under displacement control, with boundary conditions applied to the reference point at the roller center. The bottom of the tape was fixed, and the contact interface was defined using a frictionless hard contact interaction.

For a thermal insulation thickness of 1.6 mm (two layers), the FE modeling method remains consistent with that described above.

**Figure 5:**
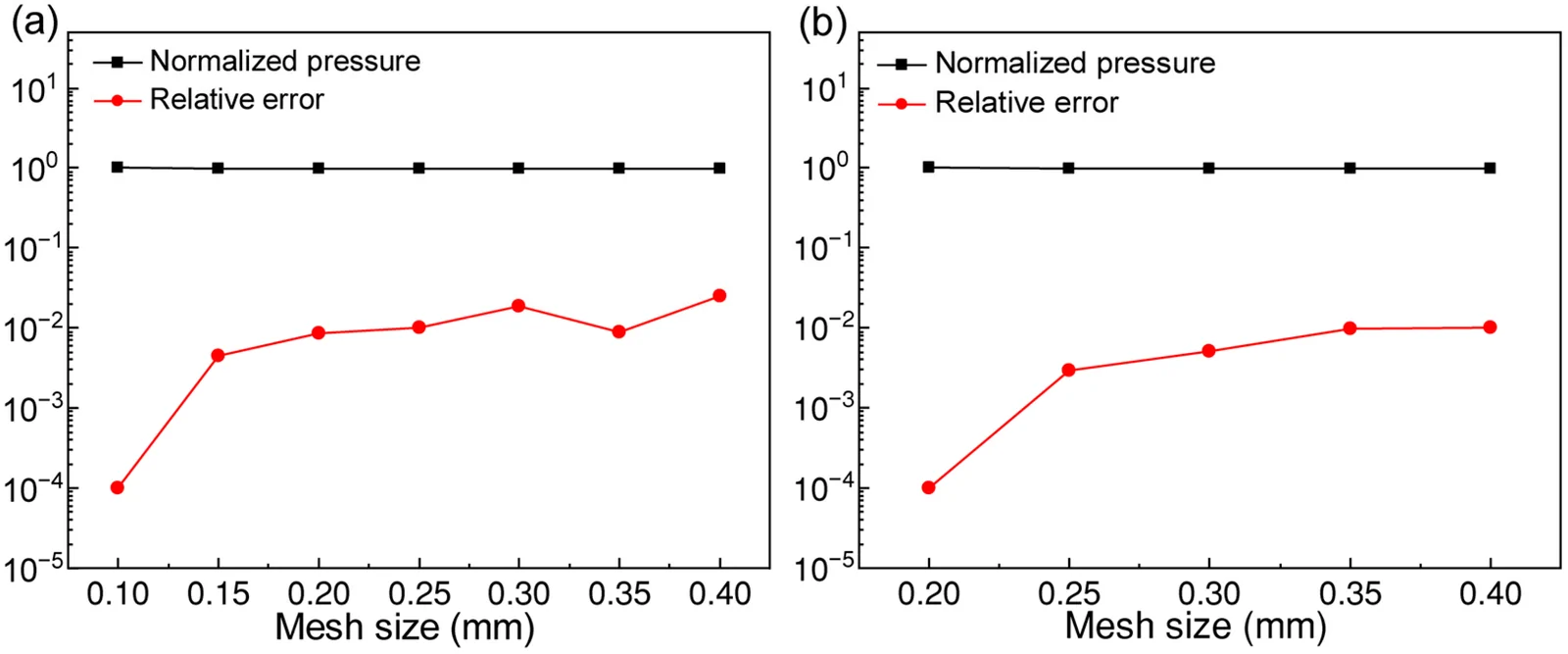
Mesh sensitivity analysis of the FE model (the relative error is shown as $\left|1-\mathrm{normalized}\;\mathrm{pressure}\right|+0.0001$, where the offset of 0.0001 is applied to avoid undefined values on the logarithmic scale): (**a**) *t* = 0.8 mm; (**b**) *t* = 1.6 mm.

The baseline mesh convergence study for the roller-tape contact was established in [4]. Building upon those findings, a sensitivity analysis was carried out focusing on the contact pressure at point *P*_m_ [5] to accommodate the ECPR and different insulation thicknesses. The element size was varied to identify the optimal discretization, ranging from 0.4 mm down to 0.1 mm for the one-layer FE model and to 0.2 mm for the two-layer model. As shown in Figure 5a,b, the pressure data were normalized by the values from the finest mesh. Since the ECPR profile is close to a straight line, the convergence curve for the one-layer model is highly similar to the cylindrical case in [4]. The results indicate that element sizes of 0.1 mm (one-layer) and 0.2 mm (two-layer) are fine enough to achieve convergence and ensure calculation accuracy.

### 3.2. Verification of the FE Model

The FE model was verified against Hertz theory [4,24].

**Figure 6:**
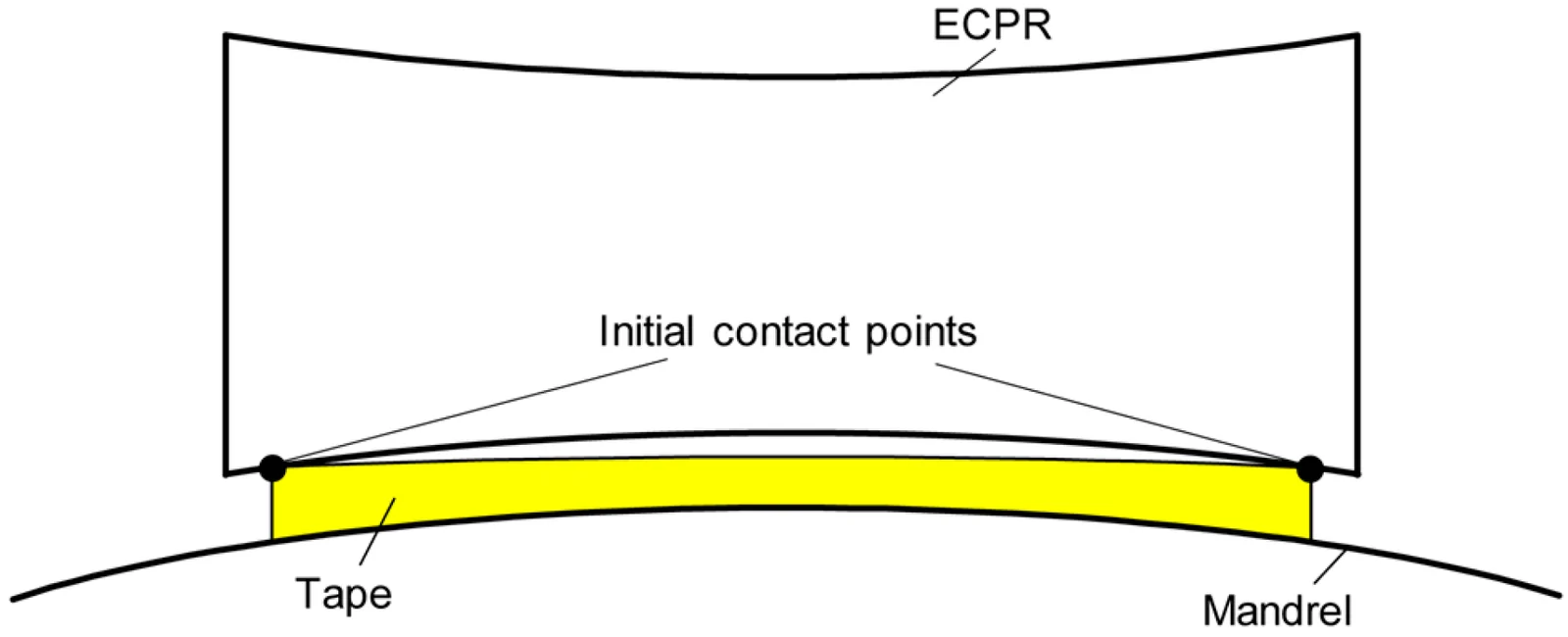
Schematic of the initial ECPR–tape contact.

As shown in Figure 6, there are two initial contact points between the ECPR and the tape, which does not satisfy the applicability conditions of Hertz theory. To address this, the ECPR shown in Figure 3a is rotated about the *z*_1_-axis so that its axis becomes perpendicular to the mandrel axis, thereby ensuring a single initial contact point. However, under such conditions, the limited thickness of the thermal insulation renders Hertz theory inapplicable when the applied displacement is large. To satisfy the theoretical requirements, the thermal insulation thickness is increased, and the applied displacement is reduced.

To incorporate these theoretical adjustments into the simulation, the one-layer FE model was modified accordingly. Specifically, the press roller axis was aligned perpendicular to the mandrel axis, the thermal insulation thickness was increased, and the applied displacement was reduced. However, the element size was maintained consistent with that of the original one-layer FE model.

**Figure 7:**
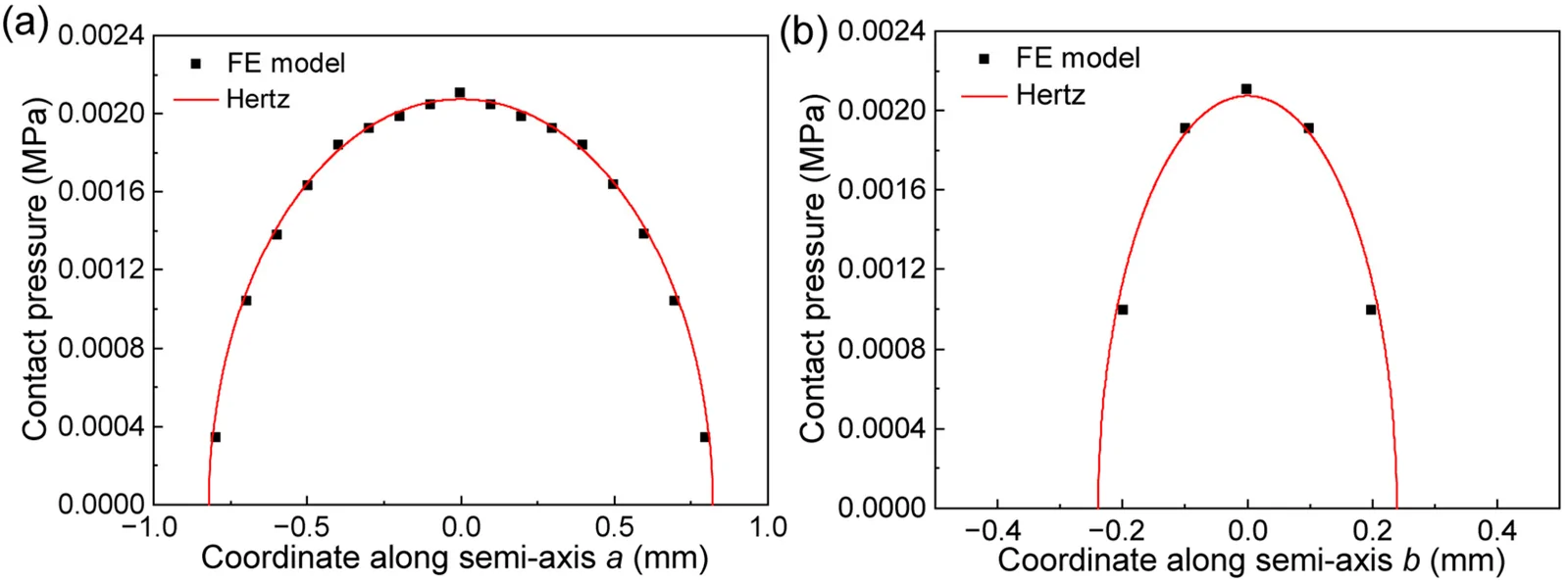
FE model and Hertz theory predictions for contact pressure distribution with parameters *R* = 240 mm, *t* = 20 mm, and *s* = 0.002 mm: (**a**) along semi-axis *a*; (**b**) along semi-axis *b*.

The verification results for the FE model with parameters *R* = 240 mm, *t* = 20 mm, and *s* = 0.002 mm are shown in Figure 7. The contact pressure distribution in the FE model agrees well with the theoretical predictions of Hertz theory, thereby confirming the validity of the simulation model. Since the one-layer FE model employs the same discretization strategy, it is considered a reliable model for simulating the ECPR–tape contact.

Furthermore, as the tape’s Poisson’s ratio decreases, the contact pressure at point *P*_m_ obtained from the one-layer FE model gradually approaches the theoretical results calculated from Equation (29) (see Section 3.3), which further confirms the model’s validity.

Since, except for the mesh size, the two-layer FE model employs the same modeling method as the one-layer FE model and converges, it can also be considered valid.

### 3.3. Determination of K_c_

In Section 2.2, the corrected elastic modulus of the foundation was not determined; here, it is obtained by fitting to the FE results [30]. Since the thermal insulation is bonded to the mandrel, the effect of this fixed boundary condition on the contact pressure varies with the thickness of the thermal insulation [23]. Therefore, the corrected elastic modulus of the foundation was calibrated specifically for thermal insulation thicknesses of 0.8 mm and 1.6 mm.

**Figure 8:**
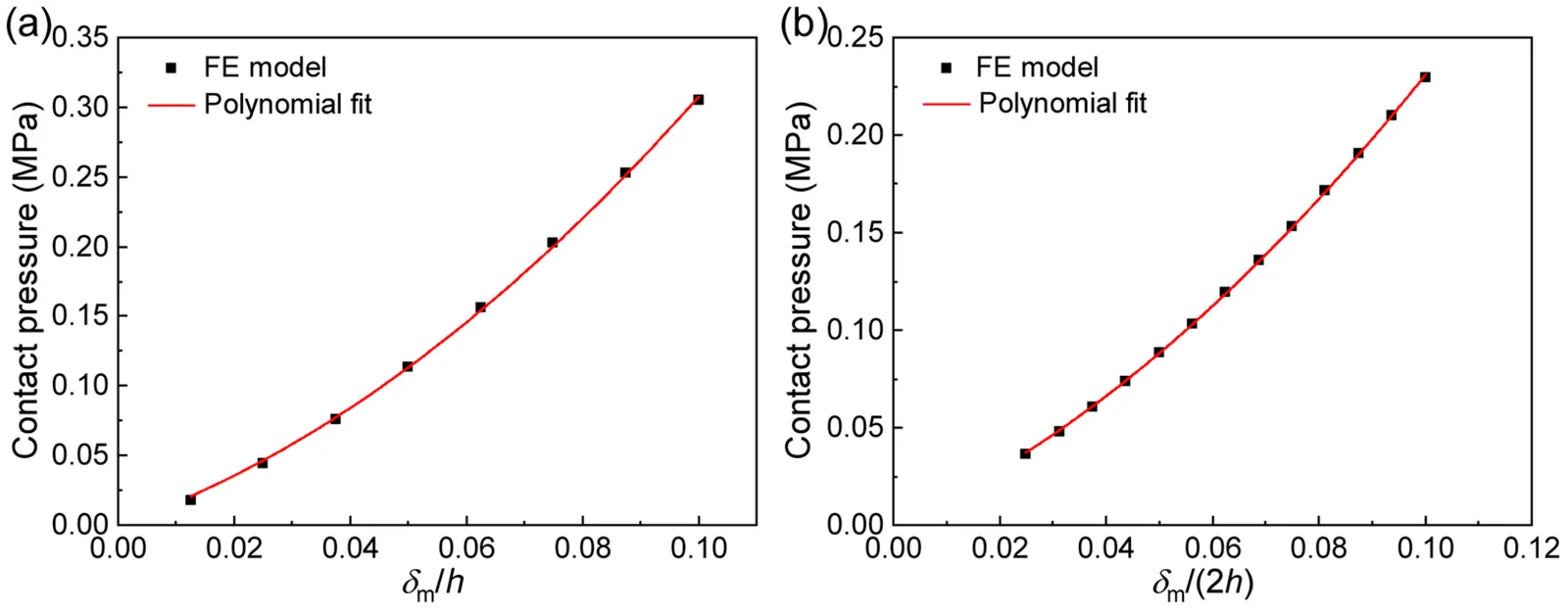
Contact pressure at point *P*_m_ as a function of *δ*_m_/*t*: (**a**) *t* = 0.8 mm; (**b**) *t* = 1.6 mm.

As shown in Figure 8, the simulation results indicate that the contact pressure at point *P*_m_ does not exhibit a linear relationship with *δ*_m_/*t*, where *δ*_m_ is the normal displacement at point *P*_m_ (*δ*_m_ = *s*). The least-squares method was used to fit a quadratic function to this relationship, thereby determining the corrected elastic modulus of the foundation.

For the thermal insulation thickness of 0.8 mm, the fitting results are shown in Figure 8a. The corresponding fitting equation is given in Equation (25).

(25) $$p_{m}=16.22{\left(\frac{\delta_{m}}{h}\right)}^{2}+1.453\left(\frac{\delta_{m}}{h}\right)$$

where *p*_m_ is the contact pressure at point *P*_m_. The corrected elastic modulus of the foundation is expressed as:

(26) $$K_{c}=16.22\left(\frac{\delta_{\max}}{h}\right)+1.453$$

For the thermal insulation thickness of 1.6 mm, the fitting results are shown in Figure 8b. The corresponding fitting equation is given in Equation (27).

(27) $$p_{m}=10.905{\left(\frac{\delta_{m}}{2h}\right)}^{2}+1.218\left(\frac{\delta_{m}}{2h}\right)$$

The corrected elastic modulus of the foundation is expressed as:

(28) $$K_{c}=10.905\left(\frac{\delta_{\max}}{2h}\right)+1.218$$

A comparison between Equations (26) and (28) indicates that, for a constant *δ*_m_, the corrected elastic modulus of the foundation is larger for the thinner thermal insulation. This can be explained by the fact that a thinner thermal insulation increases the geometric confinement imposed by the fixed rigid base. This observation aligns with the findings reported in [23].

**Figure 9:**
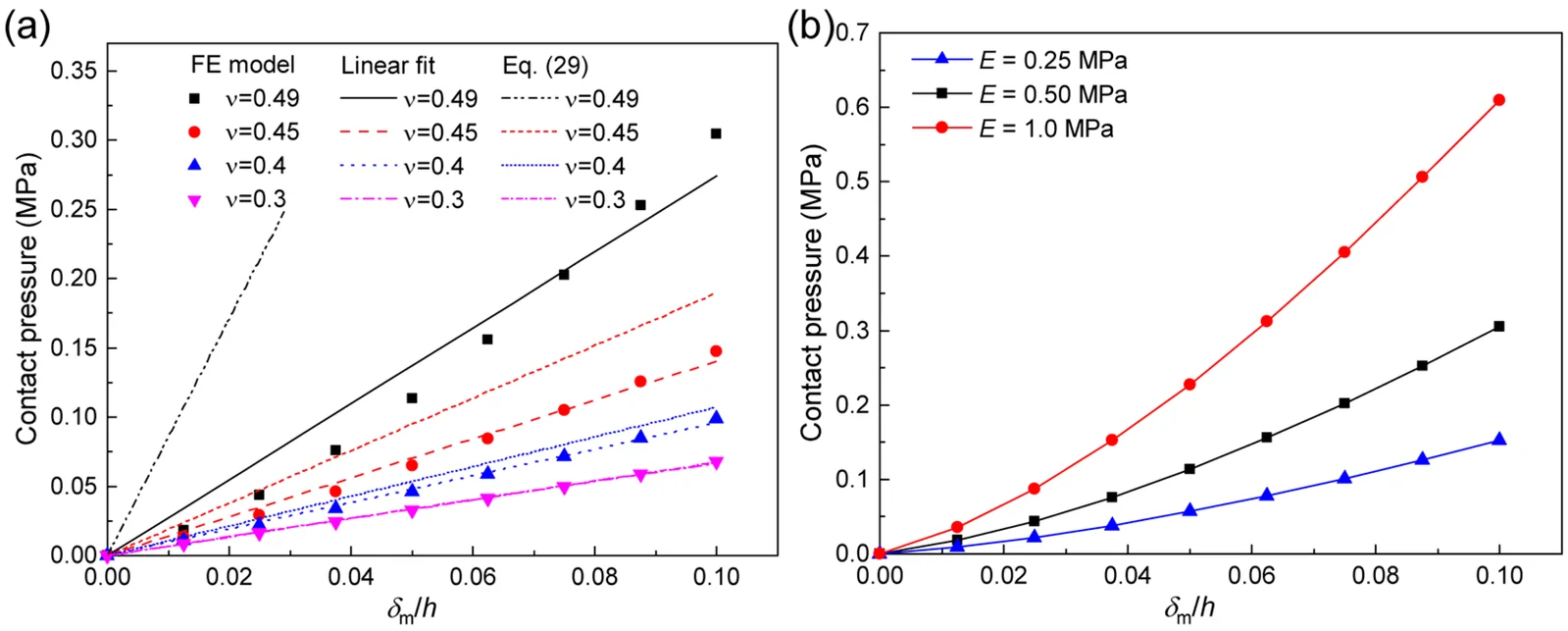
Sensitivity analysis of the contact pressure at point *P*_m_ to the tape’s elastic properties: (**a**) dependence on *δ*_m_/*h* for different Poisson’s ratios (*E* = 0.5 MPa); (**b**) dependence on *δ*_m_/*h* for different elastic moduli (*ν* = 0.49).

The nonlinear relationship between the contact pressure at point *P*_m_ and *δ*_m_/*t* is attributed to the nearly incompressible nature of the tape (*ν* ≈ 0.5). To verify this, the FE model with a thermal insulation thickness of 0.8 mm was employed. Simulations were conducted with the Poisson’s ratio of the tape set to 0.45, 0.4, and 0.3 to obtain the contact pressure at point *P*_m_ under different applied displacements. Linear fittings were performed separately for these results and the simulation data for the Poisson’s ratio of 0.49 using the least-squares method, as shown in Figure 9a. Thus, the elastic moduli of the foundation for Poisson’s ratios of 0.49, 0.45, 0.4, and 0.3 were obtained. The equation, denoted as Equation (29) in Figure 9a, is given by [11]:

(29) $$p_{m}=\frac{E\left(1-\nu \right)}{\left(1+\nu \right)\left(1-2\nu \right)}\left(\frac{\delta_{m}}{h}\right)$$

The results indicate that as the Poisson’s ratio of the tape decreases, the linearity between the contact pressure at point *P*_m_ and *δ*_m_/*h* gradually increases, with the Pearson correlation coefficient increasing from 0.9928 to 0.9999. Additionally, as the Poisson’s ratio of the tape decreases, the elastic modulus of the foundation approaches the theoretical value of $E\left(1-\nu \right)/\left[\left(1+\nu \right)\left(1-2\nu \right)\right]$. This trend is consistent with the findings reported in [22]. Furthermore, a sensitivity analysis of the elastic modulus was conducted. Based on generalized Hooke’s law, the contact pressure is proportional to the elastic modulus. To verify this, additional FE simulations were performed with the elastic modulus set to 0.25 MPa and 1.0 MPa, respectively, while maintaining a constant Poisson’s ratio of 0.49. The corresponding contact pressure at point *P*_m_ is presented in Figure 9b. The results confirm that the contact pressure scales linearly with the elastic modulus for a given applied displacement. Changing the elastic modulus does not affect the non-linearity of the relationship between the contact pressure and *δ*_m_/*h*. This confirms that the non-linearity is primarily governed by the high Poisson’s ratio.

### 3.4. Determination of λ

To determine the correction factor, the semi-minor axis of the contact area was calculated using two methods: Equation (19), which yielded *c*_e0_, and the FE simulation, which yielded *c*_f0_. For the thermal insulation thicknesses of 0.8 mm and 1.6 mm, five applied displacements were selected for each case to obtain multiple pairs of *c*_e0_ and *c*_f0_. For the 0.8 mm thickness, the applied displacements ranged from 0.02 mm to 0.06 mm in increments of 0.01 mm; for the 1.6 mm thickness, they ranged from 0.06 mm to 0.10 mm in increments of 0.01 mm. The correction factor was obtained by performing a linear least-squares fit of *c*_f0_ as a function of *c*_e0_ for each thickness.

**Figure 10:**
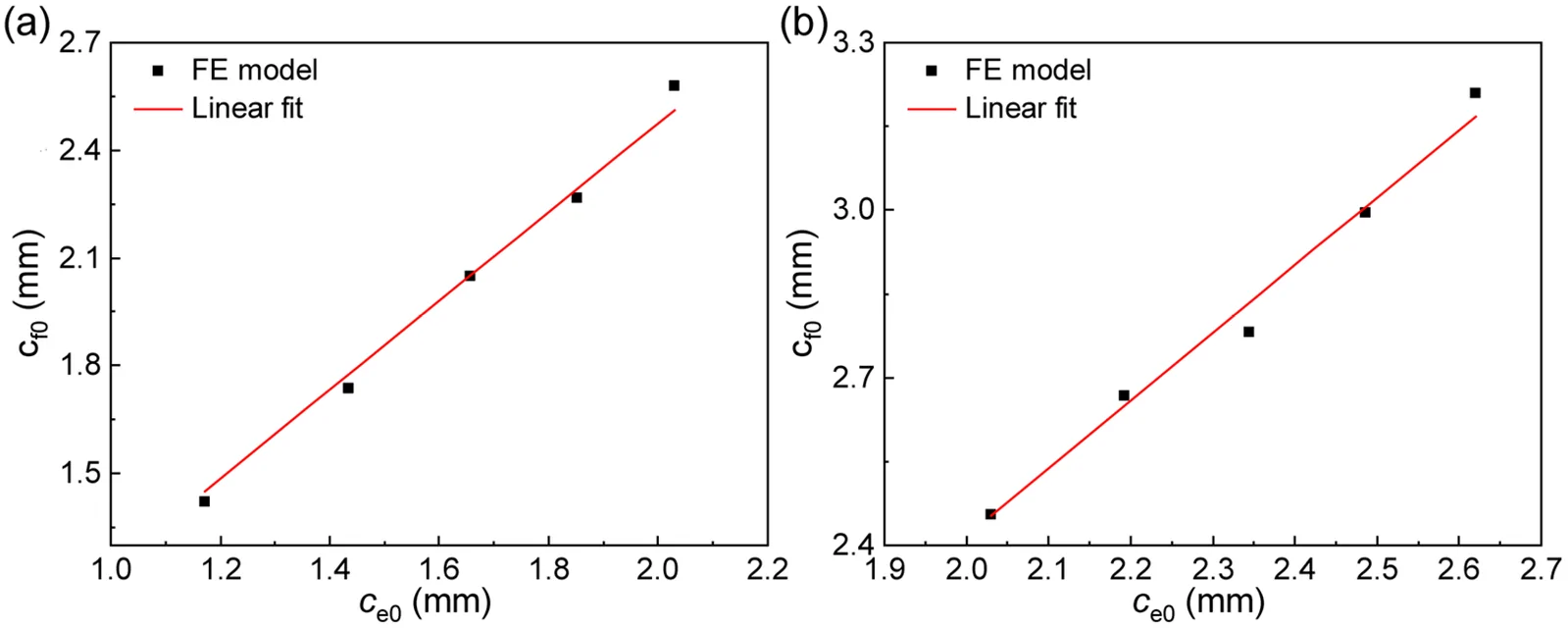
Results of linear least-squares fit of *c*_f0_ as a function of *c*_e0_: (**a**) *t* = 0.8 mm; (**b**) *t* = 1.6 mm.

The fitting results for the thermal insulation thicknesses of 0.8 mm and 1.6 mm are shown in Figure 10a and Figure 10b, respectively. The correction factor *λ* was determined to be 1.237 for the 0.8 mm thickness and 1.209 for the 1.6 mm thickness.

**Figure 11:**
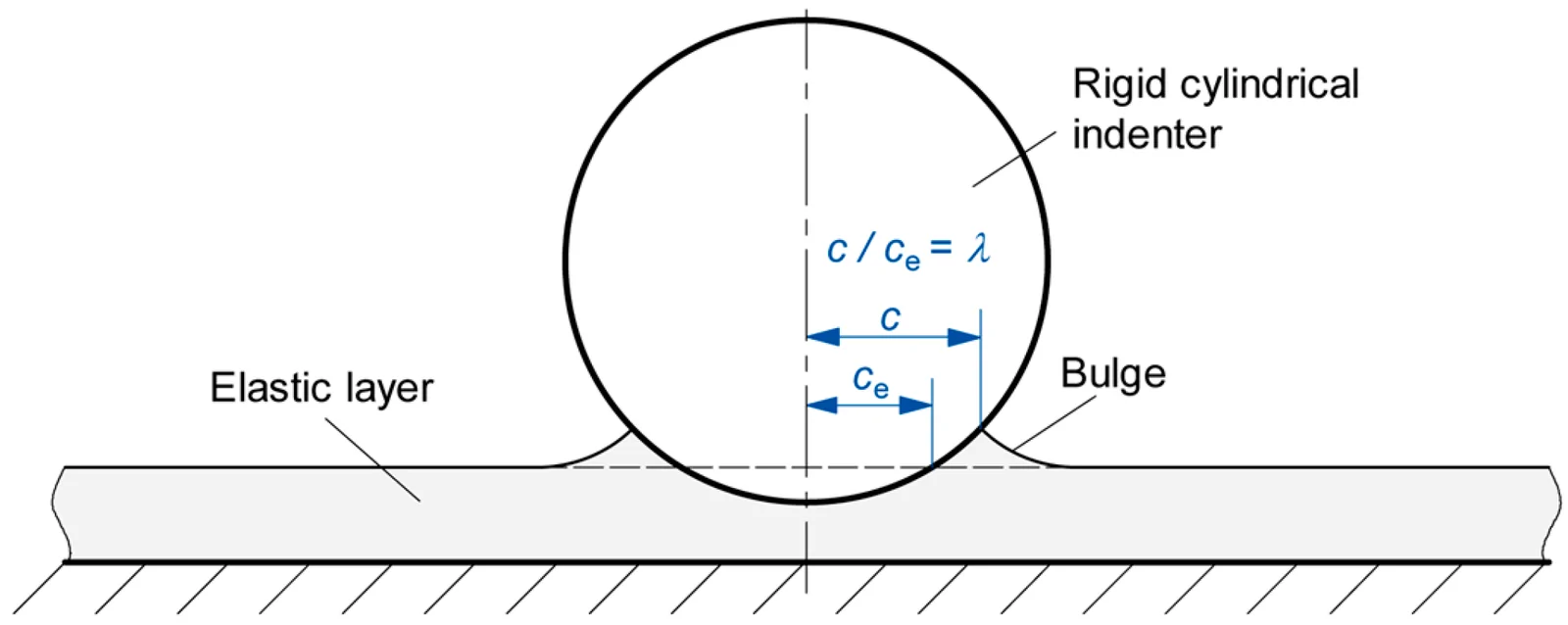
Frictionless contact between a rigid cylindrical indenter and an elastic layer bonded to a rigid substrate.

Treating the correction factor *λ* as a constant for a given contact configuration is theoretically justified. Figure 11 shows the contact problem between a rigid cylindrical indenter and an elastic layer that is bonded to a rigid substrate, assuming frictionless contact. When the contact width is comparable to or larger than the layer thickness, and the Poisson’s ratio of the layer is 0.5, theoretical analysis yields a correction factor *λ* of $\sqrt{3}$ [10]. Similarly, when the rigid cylindrical indenter in Figure 11 is replaced with a rigid spherical indenter to produce a central indentation, the theoretical value of the correction factor *λ* under the same conditions is $\sqrt{2}$ [11].

With the calibration of these parameters in Section 3.3 and Section 3.4, the proposed modeling framework is now complete. It is worth noting that while the framework is fundamentally based on classical contact mechanics, the theoretical model possesses a semi-empirical nature because its two key parameters (*K*_c_ and *λ*) are calibrated via FE simulations rather than derived analytically.

## 4. Results

### 4.1. Comparison Between Theoretical and Numerical Results

**Figure 12:**
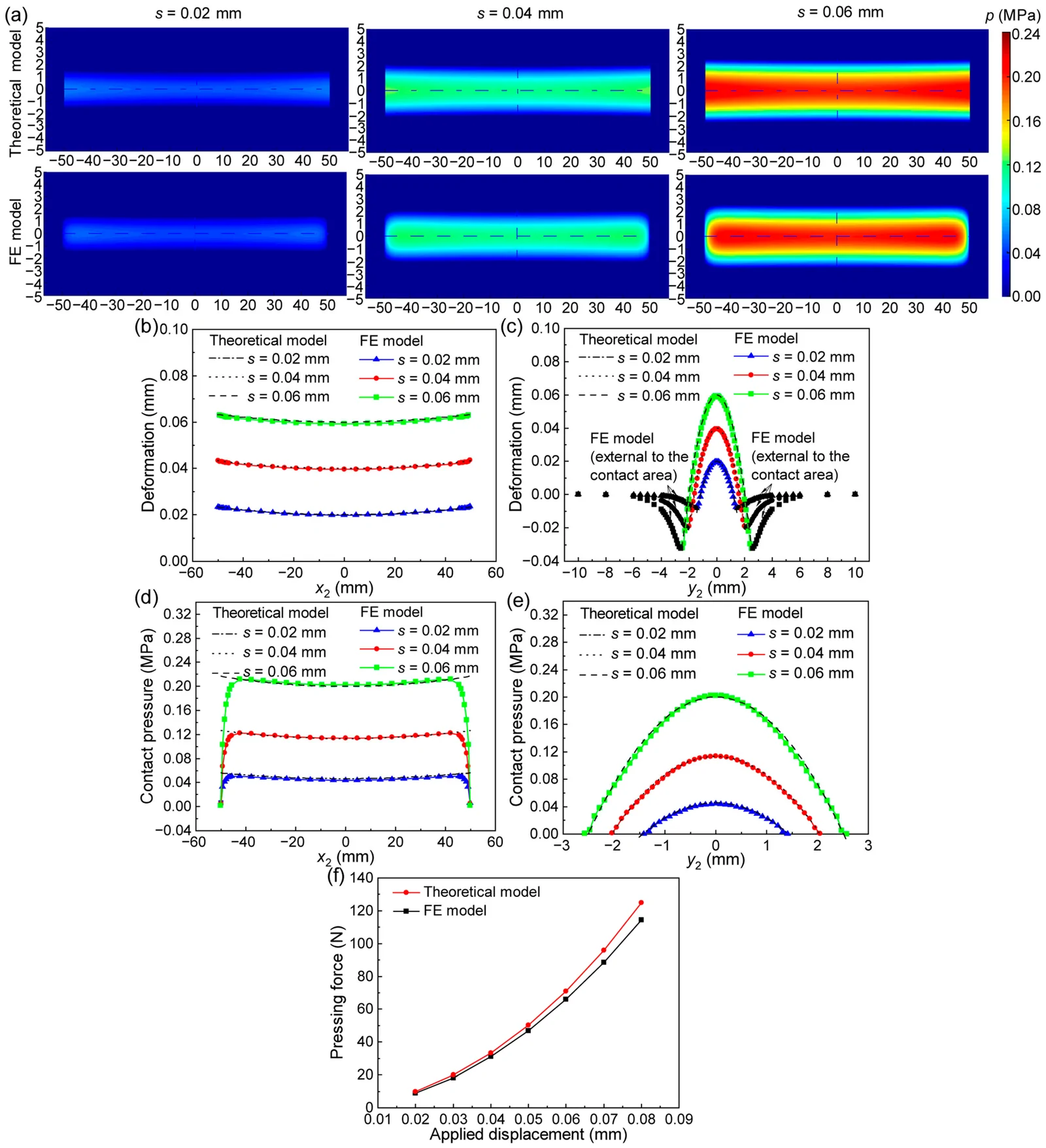
Comparison of theoretical and FE results under different applied displacements (*t* = 0.8 mm): (**a**) contact pressure distribution (major and minor axes parallel to *x*_2_- and *y*_2_-axes, respectively; dash-dotted and dotted lines denote theoretical and FE results, respectively); (**b**) deformation in the *z*_2_ direction along the major axis; (**c**) deformation in the *z*_2_ direction along the minor axis; (**d**) contact pressure distribution along the major axis; (**e**) contact pressure distribution along the minor axis; (**f**) pressing force.

**Figure 13:**
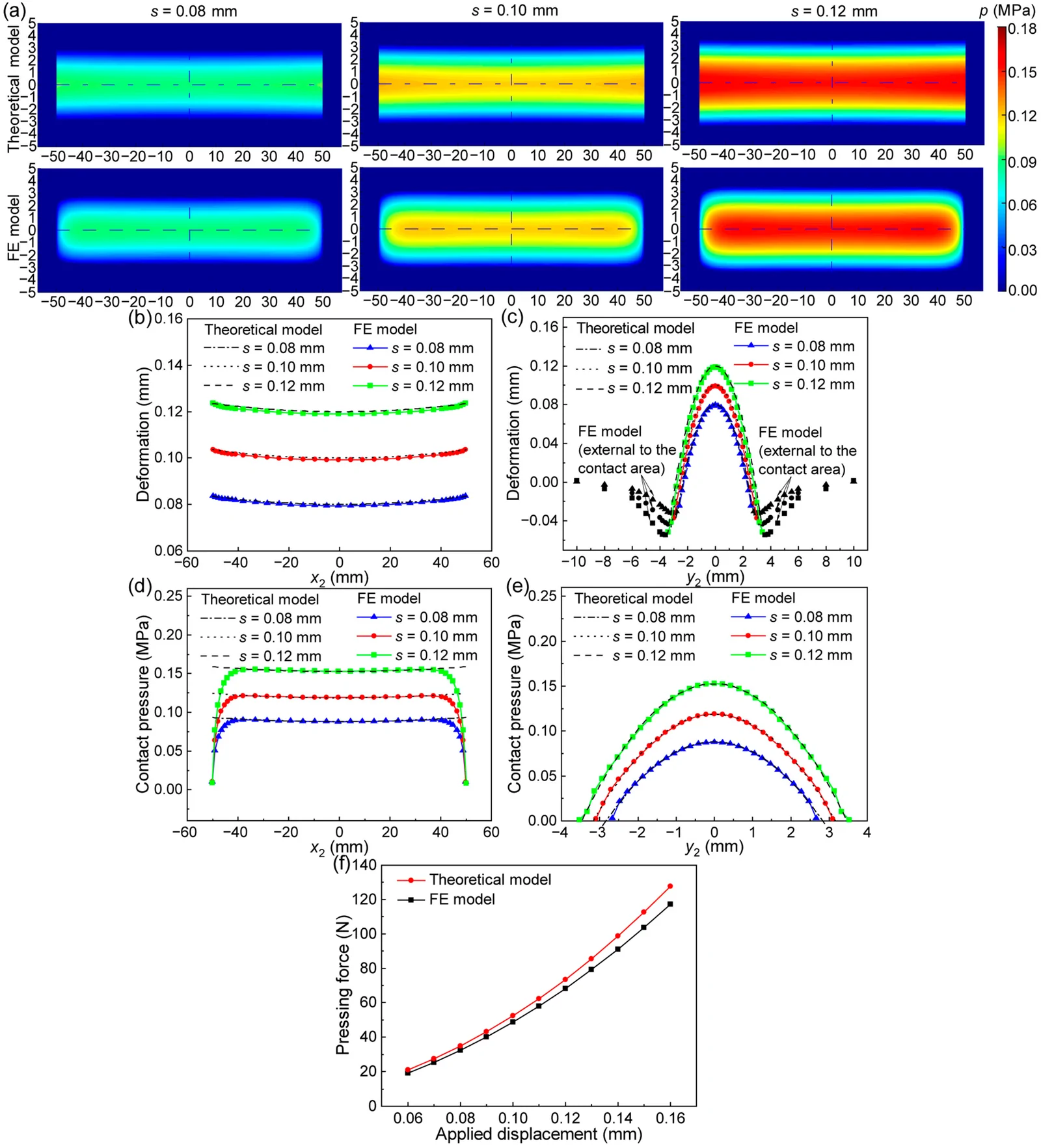
Comparison of theoretical and FE results under different applied displacements (*t* = 1.6 mm): (**a**) contact pressure distribution (see Figure 12a for axes definition); (**b**) deformation in the *z*_2_ direction along the major axis; (**c**) deformation in the *z*_2_ direction along the minor axis; (**d**) contact pressure distribution along the major axis; (**e**) contact pressure distribution along the minor axis; (**f**) pressing force.

Figure 12 and Figure 13 present the theoretical and FE results of the overall contact pressure distribution, the deformation in the *z*_2_ direction along the major and minor axes of the contact area, the contact pressure distribution along these axes, and the pressing force under different applied displacements. The thermal insulation thicknesses in Figure 12 and Figure 13 are 0.8 mm and 1.6 mm, respectively. The theoretical results are in good agreement with the FE results. Furthermore, as shown in Figure 12c and Figure 13c, the thermal insulation near the edge of the contact area exhibits pronounced bulges. This bulge effect is consistent with the findings reported in [11,22].

To further verify the generality of the theoretical model, FE models with mandrel radii of 240, 400, and 1000 mm were employed. Notably, the key parameters calibrated from the 240 mm baseline case were directly used for the 400 mm and 1000 mm theoretical predictions without recalibration. The models for the 400 mm and 1000 mm mandrels were established using the same method as for the 240 mm model detailed in Section 3.1. For these two cases, simulations were performed with the thermal insulation thicknesses of 0.8 mm and 1.6 mm. These thicknesses correspond to applied displacements of 0.06 mm and 0.12 mm, respectively.

**Figure 14:**
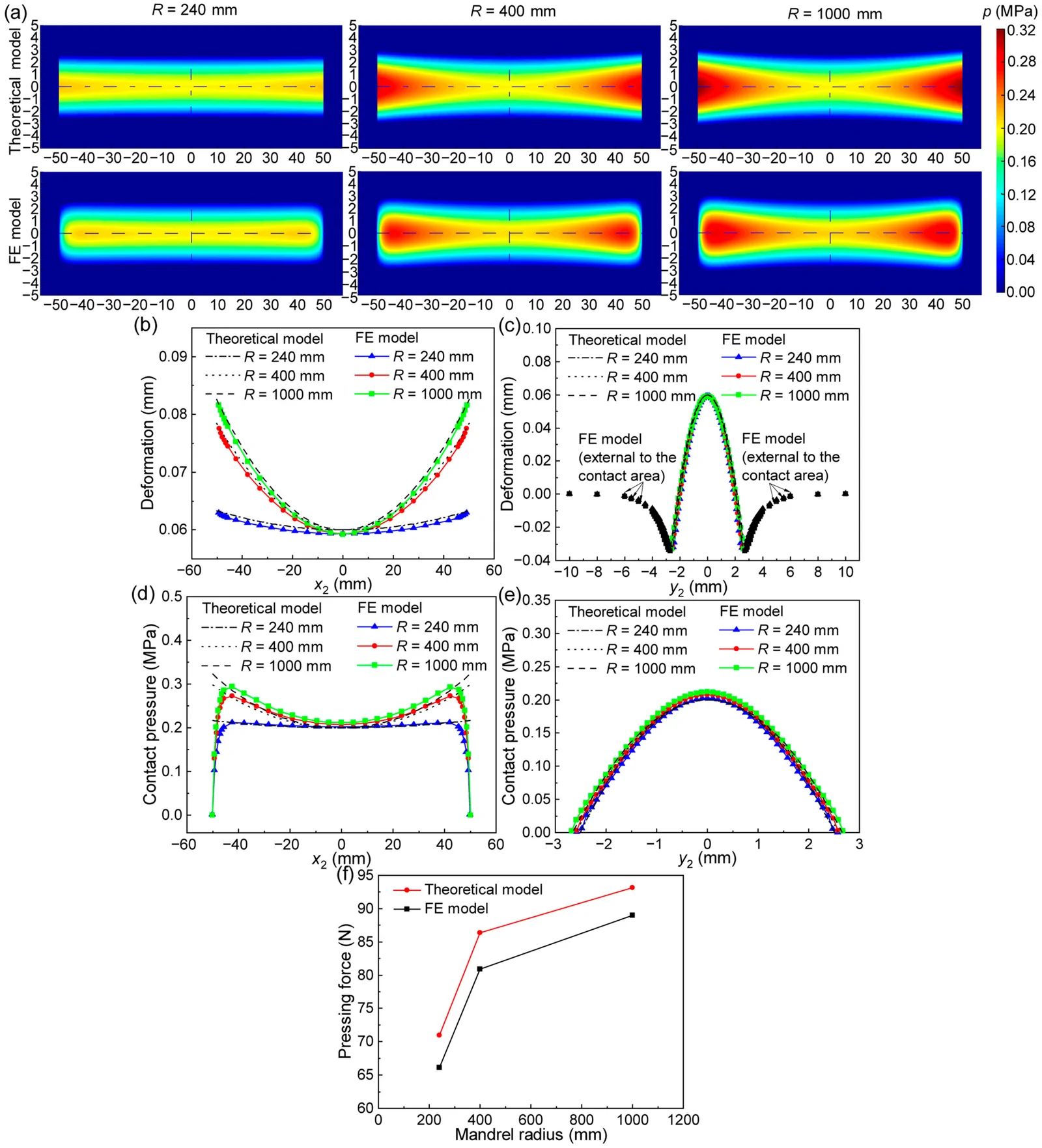
Comparison of theoretical and FE results for different mandrel radii (*t* = 0.8 mm): (**a**) contact pressure distribution (see Figure 12a for axes definition); (**b**) deformation in the *z*_2_ direction along the major axis; (**c**) deformation in the *z*_2_ direction along the minor axis; (**d**) contact pressure distribution along the major axis; (**e**) contact pressure distribution along the minor axis; (**f**) pressing force.

**Figure 15:**
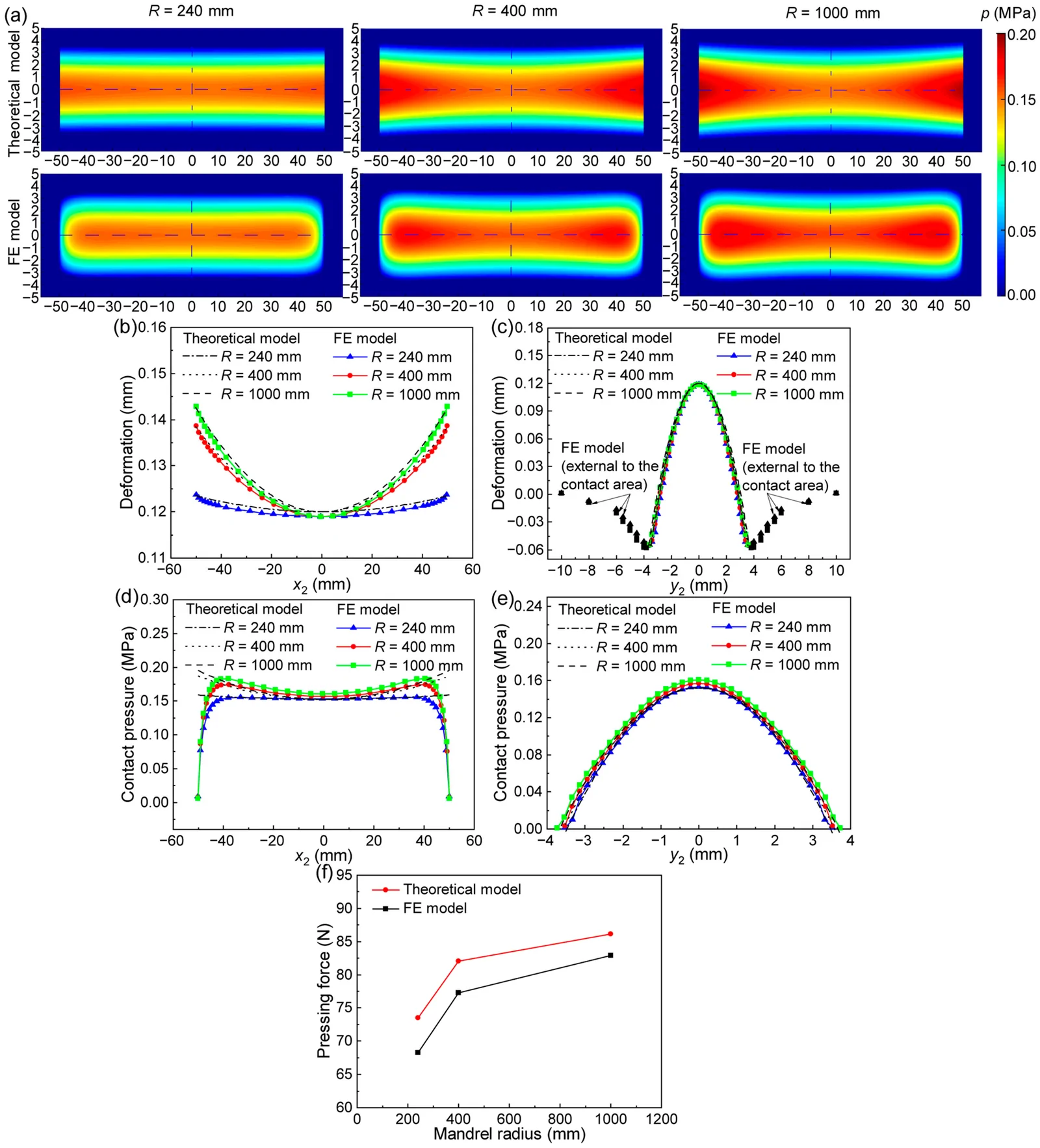
Comparison of theoretical and FE results for different mandrel radii (*t* = 1.6 mm): (**a**) contact pressure distribution (see Figure 12a for axes definition); (**b**) deformation in the *z*_2_ direction along the major axis; (**c**) deformation in the *z*_2_ direction along the minor axis; (**d**) contact pressure distribution along the major axis; (**e**) contact pressure distribution along the minor axis; (**f**) pressing force.

Figure 14 and Figure 15 compare the theoretical and FE results for different mandrel radii. The theoretical results show close agreement with the FE results, further confirming the model’s generality. As shown in Figure 14c and Figure 15c, the thermal insulation near the edge of the contact area also exhibits pronounced bulges.

### 4.2. Experimental Validation

**Figure 16:**
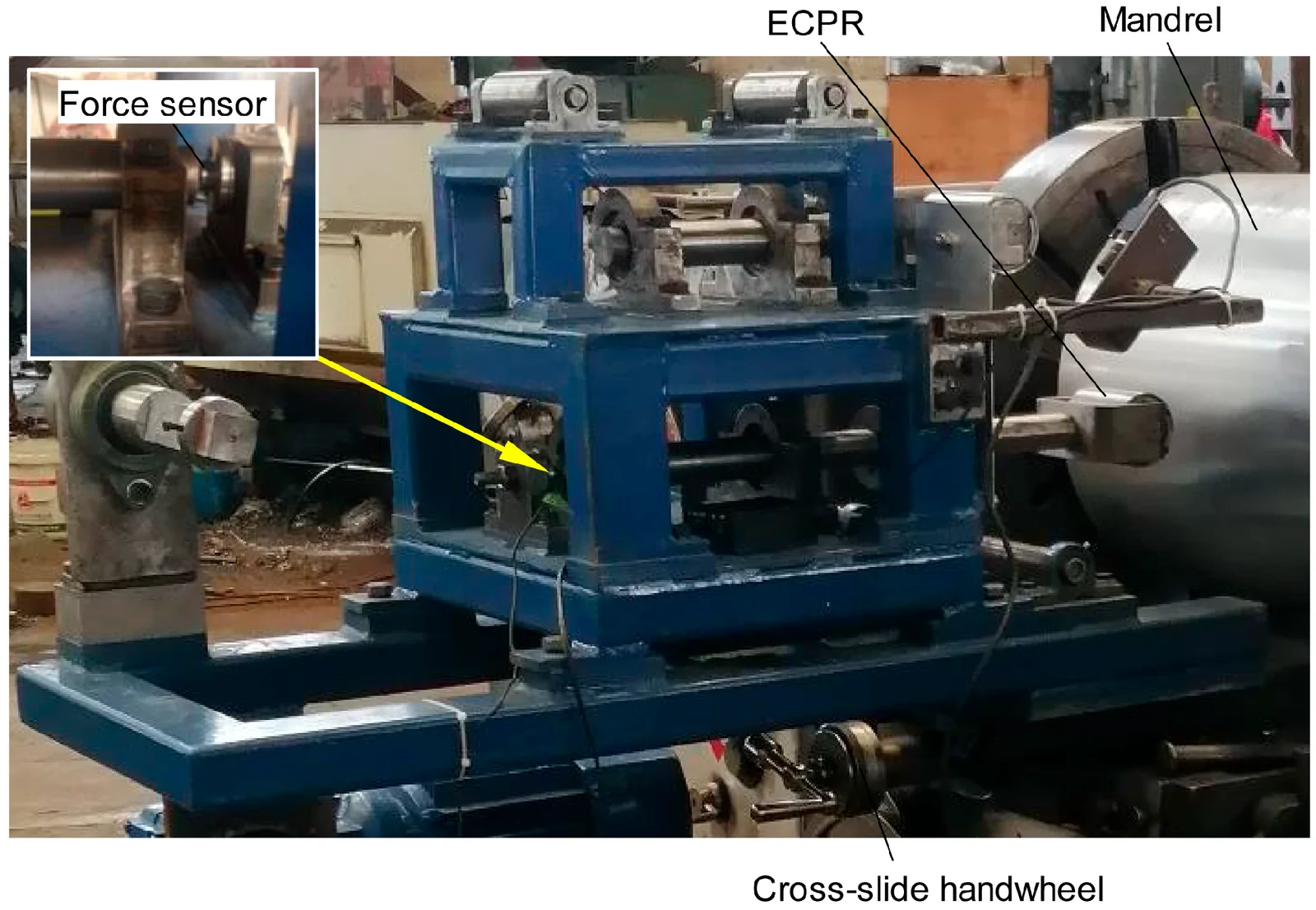
Experimental setup for pressing force measurement.

The pressing force measurement experiment was conducted to validate the theoretical model. Figure 16 shows the experimental platform, which was adapted from a lathe. The mandrel is clamped between the lathe chuck and the tailstock. The applied displacement is controlled by adjusting the cross-slide handwheel. The pressing force is measured using a force sensor installed behind the support shaft of the press roller.

In the experiment, a mandrel with a radius of 250 mm was employed. The press roller was designed with an elliptical concave surface, as outlined in Section 2.1. The length of the press roller is 98 mm, and its central cross-sectional radius is 40 mm. Both the mandrel and the press roller are made of steel. Each has an elastic modulus of 200,000 MPa and a Poisson’s ratio of 0.3. The tape used in the experiment has a thickness of 0.9 mm and a width of 96 mm. Although the target application utilizes uncured EPDM rubber tape, uncured natural rubber tape was selected for the experimental validation. This substitution is justified because both materials exhibit two fundamental constitutive behaviors: linear elasticity under low deformation levels (<10%) [21,25,26] and near-incompressibility. Therefore, their deformation behaviors can be characterized by the elastic modulus and Poisson’s ratio. Specifically, the Poisson’s ratios for both the uncured natural rubber and the uncured EPDM rubber are estimated to be 0.49 [28,29]. Furthermore, their elastic moduli are of the same order of magnitude, with values of approximately 0.5 MPa for the uncured EPDM rubber and 0.3 MPa for the uncured natural rubber. Since the proposed modeling framework is based on these fundamental constitutive behaviors and elastic properties, validating the methodology using uncured natural rubber tape demonstrates its applicability to uncured EPDM rubber tape. The elastic properties of the uncured natural rubber are to be experimentally analyzed later in this section.

The experimental procedure was conducted as follows: First, the press roller was adjusted to the winding angle calculated based on the target thickness. Second, an adhesive was applied to the mandrel to manually bond a short strip of uncured natural rubber tape as the initial layer, aligned perpendicular to the press roller axis; subsequent layers were stacked sequentially, maintaining lateral edge alignment with the preceding layers, until the insulation reached the required thickness of 3.6 mm (comprising four layers). Third, pressing force measurements were performed at controlled displacements.

Specifically, the determination of the initial contact position was constrained by the hardware limitations: the force sensor (resolution: 1 N) was reset to zero, and the roller was advanced in increments of 0.05 mm (corresponding to the scale division of the cross-slide handwheel) until the first non-zero reading was registered. This position was designated as the nominal initial point. From this point, the pressing force was recorded at six specified displacements ranging from 0.10 mm to 0.35 mm in 0.05 mm increments. These measurements were repeated three times.

To characterize the elastic behavior of the uncured natural rubber tape and compensate for the systematic error introduced by the discrete displacement steps and the sensor resolution, a hybrid inverse analysis strategy was adopted. The six experimental data points were divided into two distinct datasets: (1) a calibration set (displacements of 0.10, 0.20, and 0.30 mm) used for parameter identification, and (2) a validation set (displacements of 0.15, 0.25, and 0.35 mm) reserved for independent model verification.

A corresponding FE model was established to conduct the inverse identification based solely on the calibration set. It was recognized that the displacement offset associated with the sensor’s first non-zero reading defines the effective zero point for the measurements. Therefore, an inverse FE analysis was performed to simultaneously identify two coupled unknown parameters: (1) the elastic modulus of the uncured natural rubber tape and (2) the theoretical displacement offset required to generate a reaction force of 1 N (corresponding to the sensor’s threshold reading).

**Figure 17:**
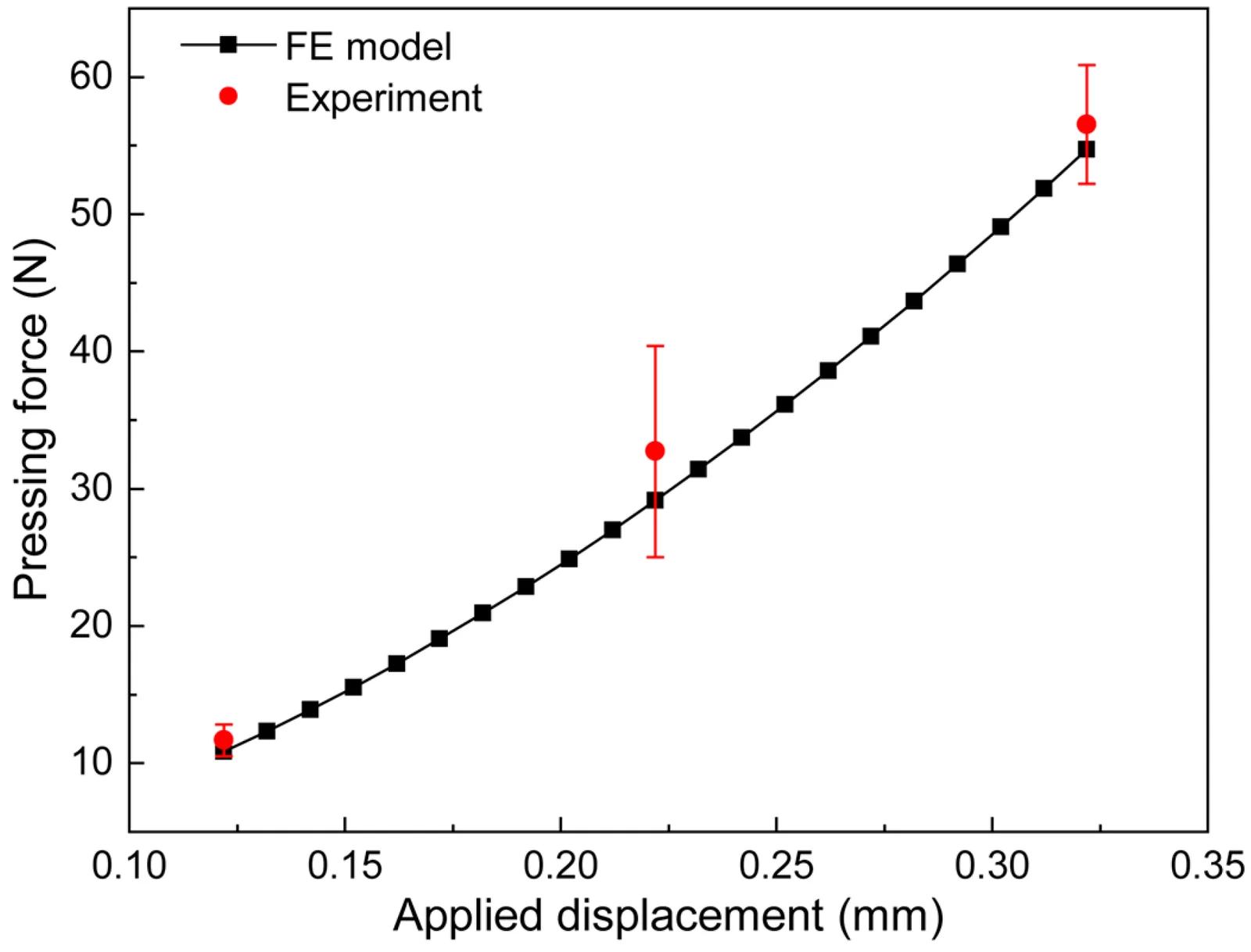
Comparison of experimental and FE load–displacement curves.

By minimizing the discrepancy between the FE predictions and the calibration set, the inverse analysis converged to an elastic modulus of 0.3 MPa and a displacement offset of 0.022 mm. This implies that a theoretical displacement of 0.022 mm is required to reach the 1 N threshold. Consequently, to align the reference points of the experiment and the simulation, the nominal experimental displacements were corrected by compensating for this 0.022 mm offset. As shown in Figure 17, the linear elastic model with these identified parameters shows good agreement with the experimental behavior, confirming the appropriateness of the material constitutive assumption [31].

To validate the theoretical model, the experimental results of the pressing force under different applied displacements were compared with the theoretical predictions. The key parameters of the theoretical model were obtained based on the elastic properties identified from the inverse FE analysis. The corrected elastic modulus of the foundation was determined following the method outlined in Section 3.3 and is expressed as:

(30) $$K_{c}=2.402\left(\frac{\delta_{\max}}{4h_{e}}\right)+0.709$$

where *h*_e_ is the thickness of the uncured natural rubber tape. The correction factor *λ* was determined to be 1.139, following the procedure described in Section 3.4.

**Figure 18:**
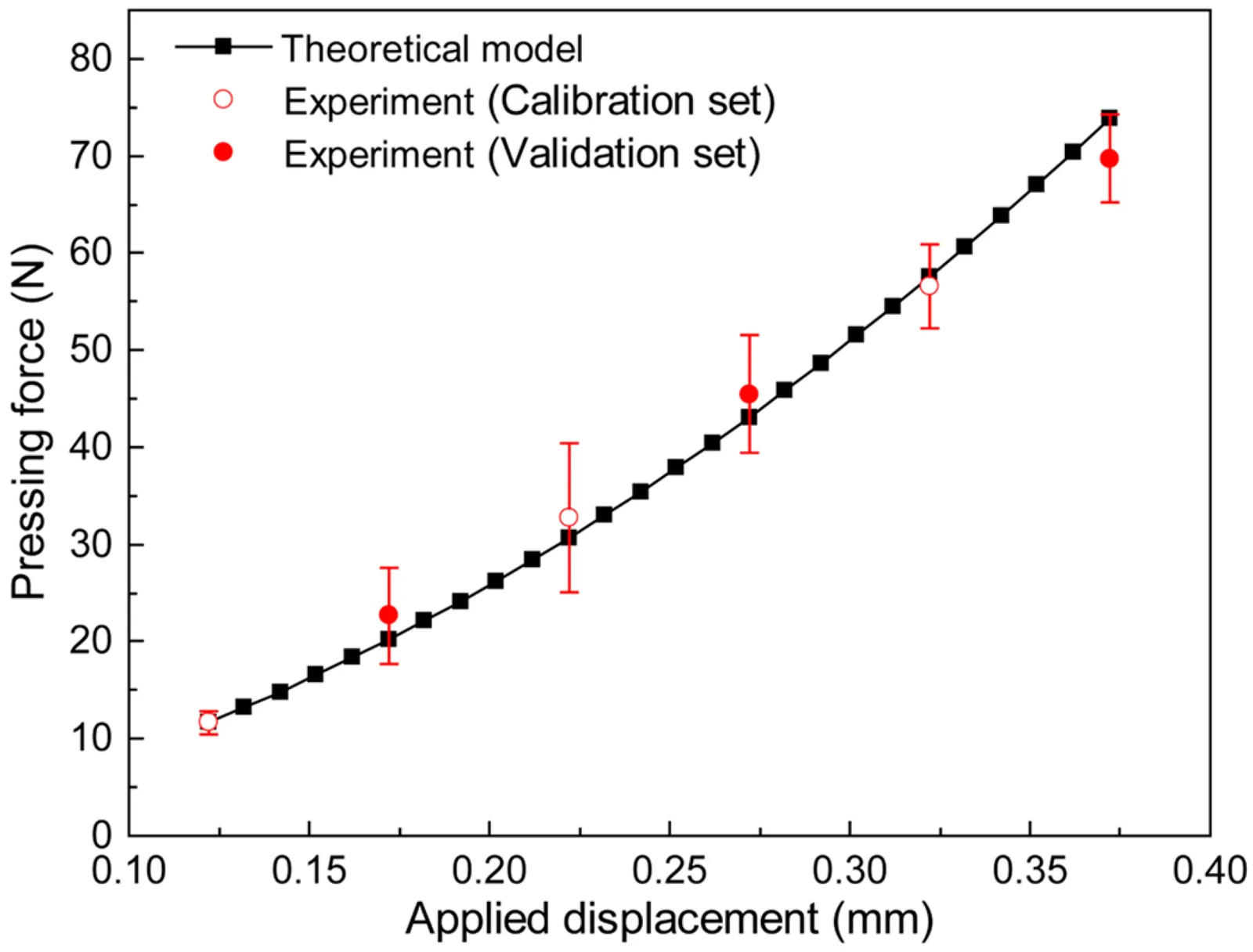
Comparison of theoretical and experimental results of pressing force under different applied displacements.

Figure 18 presents the comparison between the theoretical predictions and the experimental results. The results demonstrate close agreement across the investigated displacement range. Importantly, the theoretical model not only reproduces the responses of the calibration set but also accurately predicts the pressing forces for the validation set, which was not utilized in the parameter identification process. The relative error between the theoretical predictions and experimental results remains within 11%. Furthermore, as presented in Section 4.1, the simulated deformation, contact pressure distribution, and pressing force are also in good agreement with the theoretical predictions. Consequently, the theoretical model developed in this study is cross-validated by both numerical and experimental methods and can be reliably used to predict the contact pressure distribution.

## 5. Conclusions

This study addressed the challenges in predicting contact pressure during the thermal insulation winding process, specifically focusing on the complex three-dimensional contact between the ECPR and the thin, nearly incompressible rubber tape. The main conclusions of this study are summarized as follows:

- A novel theoretical model was developed, extending the classical EFM by incorporating correction strategies to account for material incompressibility and geometric confinement. Key parameters of the model were calibrated using FE simulations and justified through parametric and theoretical analyses, thereby establishing a reliable framework for predicting contact behavior.
- Comparisons demonstrate that the theoretical predictions of deformation, contact pressure distribution, and pressing force are in good agreement with the FE results under different applied displacements and mandrel radii without parameter recalibration, verifying the model’s generality. The global accuracy of the model was further confirmed by experimental validation employing a hybrid inverse analysis, showing a relative error of less than 11% between the predicted and measured pressing forces.
- The theoretical model provides a theoretical basis for pressure control in the winding process. Beyond the specific application, the proposed theoretical modeling strategy provides a valuable reference when solving contact problems for thin elastic layers characterized by high Poisson’s ratios (≥0.45).

Despite these contributions, a limitation of this study is that the local contact pressure distribution was not directly measured due to experimental constraints. Additionally, the theoretical model neglects viscoelastic and frictional effects. During the dynamic winding process, the inherent viscoelasticity of the rubber tape could introduce time-dependent behaviors such as stress relaxation, while roller-tape friction restricts the lateral deformation at the contact surface. Both factors could influence the actual contact pressure distribution. Future work should therefore focus on conducting direct pressure measurements and accounting for these viscoelastic and frictional effects to further refine the theoretical model for practical manufacturing conditions.

## Figures and Tables

**Table 1:** Elastic properties of the mandrel, ECPR, and tape [4].

Component	Elastic Modulus (MPa)	Poisson’s Ratio
Mandrel	1,000,000	0.1
ECPR	200,000	0.3
Tape	0.5	0.49

## Data Availability

The data presented in this study are available on request from the corresponding author due to the confidentiality requirements of the funded project.
